# Renal tubular acidosis in hereditary transthyretin amyloidosis (ATTRv)

**DOI:** 10.1590/2175-8239-JBN-2024-0016en

**Published:** 2024-07-22

**Authors:** Priscilla Cardim Fernandes, Moises Dias da Silva, Marcia Waddington-Cruz, Carlos Perez Gomes

**Affiliations:** 1 Universidade Federal do Rio de Janeiro, Hospital Universitário Clementino Fraga Filho, Centro de Estudos em Paramiloidose Antônio Rodrigues de Mello, Rio de Janeiro, RJ, Brazil.; 2 Universidade Federal do Rio de Janeiro, Hospital Universitário Clementino Fraga Filho, Divisão de Nefrologia, Rio de Janeiro, RJ, Brazil.; 3 Universidade Federal do Estado do Rio de Janeiro, Escola de Medicina e Cirurgia, Rio de Janeiro, RJ, Brazil.

**Keywords:** Amyloid Neuropathies, Familial, Amyloidosis, Familial, Acidosis, Renal Tubular, Prealbumin, Urinalysis

## Abstract

**Introduction:**

Hereditary transthyretin amyloidosis (ATTRv) is a severe autosomal dominant systemic disease. It affects the peripheral and autonomic nervous systems, heart, kidneys, and eyes. Amyloid deposition has been demonstrated in the glomerular and tubulointerstitial compartments of the kidney. Therefore, urinary acidification disorders such as renal tubular acidosis (RTA) may be early manifestations of renal involvement in this population.

**Objective:**

To evaluate the prevalence of RTA in individuals with ATTRv.

**Methods:**

We included symptomatic and asymptomatic individuals with TTR mutation, older than 18 years, GFR >45 mL/min/1.73m^2^, without systemic metabolic acidosis. Urinary acidification protocol was performed with furosemide and fludrocortisone after 12 h of water deprivation (water deprivation test - WDT) and measurements of urine ammonium ( ${\text{UNH}}_{4}^{+}$ ) and titratable acidity (UTA). Proximal RTA (pRTA) was diagnosed when FEHCO^3^>10%. Incomplete form distal RTA (dRTA) was diagnosed if UpH>5.3.

**Results:**

We selected 49 individuals with a mean age of 40 (35.5–56.5) years, 63% of which were female, 84% were Caucasian, and mean GFR was 85.5 ± 20.5 mL/min/1.73m^2^. 94% had the genetic variant Val50Met and 57% were symptomatic. The prevalence of pRTA was 2% and of dRTA was 16.3%. In the subgroup with dRTA, there was no significant increase in excretion of ${\text{UNH}}_{4}^{+}$ and UTA. We observed a good correlation between UpH by potentiometry and UpH dipstick. A UpH<5.5 on the dipstick had 100% sensitivity and negative predictive value to exclude dRTA.

**Conclusion:**

A high prevalence of RTA was found in individuals with TTR mutations. The UpH dipstick after WDT had good accuracy for screening for dRTA. Further studies are needed to evaluate the impact of early diagnosis and treatment of RTA in this population.

## Introduction

Hereditary transthyretin amyloidosis (ATTRv) is an autosomal dominant disease with variable penetrance and is the most common form of hereditary amyloidosis. There are currently about 120 different mutations that produce mutant TTR, of which only about ten types are non-pathogenic, and Val50Met is the most common mutation^1^.

ATTRv is a multisystem, progressive, and disabling disease. The clinical presentation is characterized by involvement of the peripheral nervous system, heart, digestive system, kidneys, and eyes. The clinical presentation is variable, ranging from exclusive neurological involvement to sporadic cases with strictly cardiologic manifestations^2^. The classic course of ATTRv consists of sensorimotor and autonomic polyneuropathy, gastrointestinal dysfunction, cardiac conduction block, infiltrative cardiomyopathy (familial amyloid cardiomyopathy), nephropathy, and less commonly, ocular deposition leading to vitreous opacities or glaucoma^3^. The median age of onset is the fourth and fifth decades. Without treatment, the median time from symptom onset to death is approximately 10 to 15 years^4^.

Initially considered a benign disease in terms of renal function, it was later recognized that progression to end-stage renal disease occurs as a natural history in up to one third of patients carrying the Val50Met mutation^5^. Nephropathy is more common in women, in patients with late-onset neuropathy, in cases with low family penetrance, and in patients with preexisting cardiomyopathy^6^. A critical issue in ATTRv nephropathy is the recognition of the presence and distribution of renal amyloid deposition. The glomeruli, medulla, basement membrane of the distal tubules, loops of Henle, and interstitium are typically filled with amyloid deposits, even in the early stages of the disease. Renal dysfunction and proteinuria are proportional to the degree of amyloid deposition in the glomeruli, arterioles, and medium vessels. However, they do not correlate with deposits in the renal medulla^7^. Currently, the diagnosis of amyloid nephropathy can be inferred from the presence of albuminuria in patients with documented neuropathy, although the gold standard for definitive diagnosis is renal histopathology. However, albuminuria is not a specific marker of tubulointerstitial damage and may be absent in about 10% of patients who progress to end-stage renal disease^8,9^. To date, tubulopathies have not been described in ATTRv.

Renal tubular acidosis (RTA) is a tubular dysfunction and consists of an inability to reabsorb filtered HCO_3_ and/or excrete an acid load through the kidneys, compromising the maintenance of acid-base balance^10^. It is distinguished from uremic acidosis by the presence of a preserved or slightly decreased glomerular filtration rate and by being a hyperchloremic acidosis with a normal anion gap^11^. There are five types of RTA: Type 2 or proximal RTA (pRTA) is characterized by impaired reabsorption or regeneration of HCO_3_ in the proximal tubule, which lowers the HCO_3_ reabsorption set point and increases the HCO_3_ excretion fraction (>10%) even with a normal serum level, presenting normokalemia/hypokalemia^12^. Classical type 1 or distal RTA (dRTA) is characterized by impaired distal acidification of urine, with no decrease in urine pH in the presence of acidemia or even after acid overload^13^, and is also characterized by normokalemia or hypokalemia. On the other hand, voltage-dependent type 1 or distal RTA is characterized by hyperkalemia. Type 3 RTA is rare and presents characteristics of both distal and proximal forms. Type 4 or hyperkalemic RTA is caused by aldosterone deficiency or tubular resistance to this hormone^14^.

Therefore, it is pertinent to evaluate whether the affected population has a urinary acidification disorder, since this tubular dysfunction is possible due to medullary deposits and could be a valuable tool in early diagnosis of renal involvement in ATTRv. The aim of this study was to analyze the prevalence of RTA in patients with ATTRv and to compare different subgroups of patients according to the diagnosis of RTA and the presence of extrarenal symptoms.

## Methods

A cross-sectional study was designed to evaluate the prevalence of subclinical urinary acidification disorders in individuals with TTR mutation followed at the Antonio Rodrigues de Mello Paramyloidosis Study Center (CEPARM), located at the Clementino Fraga Filho University Hospital (HUCFF) of the Federal University of Rio de Janeiro – UFRJ, Brazil. This study was approved by the local ethics committee of HUCFF-UFRJ (Project number 47772021.5.0000.5257), and was conducted in accordance with the Declaration of Helsinki.

We included asymptomatic and symptomatic individuals older than 18 years with eGFR > 45 mL/min/1.73m^2^ estimated by the CKD-EPI (Chronic Kidney Disease Epidemiology Collaboration) equation and preserved voiding control. We excluded patients with active urinary tract infection, HIV (human immunodeficiency virus), HBV (hepatitis B virus), or HCV (hepatitis C virus) infection, chronic lung disease, acute diarrhea, autoimmune diseases, pregnant women, and patients with contraindications to the use of furosemide or fludrocortisone. Patients were evaluated between March 2019 and February 2020.

We considered symptomatic patients to be those with symptoms related to sensorimotor polyneuropathy, dysautonomia, cardiac conduction disorders, arrhythmias, or systolic or diastolic dysfunction. Asymptomatic patients were those with a positive genetic test for the mutation but without any of the above symptoms or signs^1,2,3^.

All patients had their last meal at 8:00 p.m. of the previous day and remained in complete fast from liquids and solids until 8:00 a.m. to ensure maximum urine concentration for 12 hours (water deprivation test - WDT). At 8:00 a.m., a sample of the first urine was collected (baseline sample) for the following measurements: urine pH (UpH), pCO_2_, and urinary bicarbonate (UHCO_3_) by urine gas analysis (potentiometry), ammonium ( ${\text{UNH}}_{4}^{+}$ ) by spectrophotometry, titratable acid (UTA) by NaOH in burette, and urinalysis by dipstick. Fresh urine samples were collected and analyzed immediately to avoid loss of CO_2_. A venous blood sample was also taken at this initial time point for blood gas analysis and subsequent laboratory tests: complete blood count, glucose, glycated hemoglobin (HbA1c), sodium, potassium, calcium, magnesium, phosphate, chloride, uric acid, albumin, urea, creatinine, aspartate (AST) and alanine (ALT) aminotransferases, alkaline phosphatase (ALP) (45-129 U/L), and 25-hidroxy-vitamin D (25(OH)D3) (>30 ng/mL). For patients who did not achieve a baseline urine pH < 5.5 by potentiometry after WDT, we used a modified protocol described by Wash et al. to evaluate the reduced urinary acidification capacity of the distal nephron^11^. This acidification stress test consists of the administration of 40 mg furosemide and 0.1 mg fludrocortisone, followed by urine collection every hour for 4 hours for pH measurement (0, 1, 2, 3, and 4 h). In the last urine sample (4 h), ${\text{UNH}}_{4}^{+}$ and UTA were also measured for comparison with the baseline sample. pRTA was defined as FEHCO_3_ > 10%^15^. Incomplete dRTA was defined by UpH > 5.3 on all measurements and no increase in ${\text{UNH}}_{4}^{+}$ (26-28 mEq/min/1.73m^2^) or UTA (22-47 mEq/min/1.73m^2^)^16^ after the urine acidification test^11,17^.

In the same week, patients had a 24-hour urine collection for measurement of proteinuria (<150 mg/24 h), albuminuria (< 30 mg/24 h), creatinine, sodium, potassium, calcium, magnesium, phosphate, chloride, uric acid, and citrate (>320 mg/24 h)^18,19^. From the blood and urine samples we were able to calculate serum anion gap [AG = Na – (Cl + HCO_3_)] (8–14 mmol/L), urine anion gap [UAG = (Na + K) – Cl)] (negative values in mmol/L), measured GFR (> 60 mL/min/1.73m^2^), protein nitrogen appearance for estimation of protein intake [(PNA = ((urinary urea × 0. 46) + 2) × 6.25)/body weight))] (0.8–1.2 g ptn/kg/24 h), NaCl intake estimation (g/24h), and fractional excretion (FE) of electrolytes^11,12^. The FE of each electrolyte is calculated by the formula:

$$\mathrm{FE electrolyte = 100 \times}\frac{\mathrm{Urine electrolyte \times Plasma creatinine}}{\mathrm{Plasma electrolyte \times Urine creatinine}}$$

We considered the following reference values for fractional excretion: FE Na < 1%, FE K < 30%, FE Ca < 3%, phosphate reabsorption rate (PRR) [(1 - FE phosphate) × 100] > 80%, FE Mg < 6%, FE uric acid < 10%^12,20^.

Categorical variables were expressed as absolute numbers and percentages, and continuous variables were expressed as mean ± standard deviation or median (interquartile interval). Unpaired t-test or Mann-Whitney test was used for two-group comparisons, and Friedman test with post-hoc analysis was used for comparisons between three or more groups. Spearman’s correlation and linear regression analysis were used to measure the association between two variables. Receiver operator characteristic (ROC) curve was used to evaluate the accuracy of dipstick pH for dRTA diagnosis. Statistical analyses were performed using IBM SPSS Statistics, version 24 (USA), and the significance level was set at 0.05 (two-tailed). The study was approved by the local ethics committee and the subjects gave their informed consent.

## Results

At the time of recruitment, 272 patients were being treated in the outpatient clinic of CEPARM. Of these, 96 were excluded because they lived outside the state of Rio de Janeiro, 79 refused to participate, 25 could not be contacted by phone, 9 were participating in a double-blind study at the time of evaluation, 8 had an eGFR < 45 mL/min/1.73m^2^, 5 had non-TTR amyloidosis, and 1 was pregnant. Therefore, we selected 49 patients, of whom 28 were symptomatic (57.1%) and 21 were asymptomatic (42.9%). Of all symptomatic patients, 9 had previously undergone liver transplantation and 6 were taking tafamidis, a specific drug that stabilizes the mutant TTR protein.

Data are presented for the total population, by subgroups based on the presence or absence of systemic symptoms (cardiac and/or neurological), and by subgroups with and without dRTA. Baseline clinical and laboratory characteristics of the symptomatic and asymptomatic groups are shown in Tables 1, 2, and 3. Table 4 presents data exclusively from the dRTA patients. One of the patients was unable to collect 24-hour urine due to advanced urinary incontinence.

**Table 1:** Epidemiological and clinical features

	TOTAL (n = 49)	No dRTA (n = 41)	dRTA (n = 8)	“p” value	Asymptomatic (n = 21)	Symptomatic (n = 28)	“p” value
Age (yo)	40 (35.5–56.5)	47 (35.5–58.5)	40 (30.0–48.0)	0.550	37.0 (29.0–49.5)	49.0 (37.3–60.0)	**0.010**
Female sex – N (%)	31 (63.3)	27 (65.9)	4 (50)	0.395	16 (76.2)	15 (53.6)	0.108
Skin color – N (%)							
White	41 (83.7)	34 (83.0)	7 (87.5)	0.755	20 (95.2)	21 (75.0)	0.061
Non-white	8 (16.3)	7 (17.0)	1 (12.5)	0.755	1 (4.8)	7 (25.0)	0.061
BMI (kg/m^2^)	24.9 ± 4.9	25.1 ± 4.6	25.1 ± 5.3	0.580	26.4 ± 4.8	24.0 ± 4.8	0.087
Mutation – N (%)							
Val50Met	46 (93.9)	38 (92.7)	8 (100)	0.435	19 (90.5)	27 (96.4)	0.399
Other	3 (6.1)	3 (7.3)	0 (0)	0.435	2 (9.5)	1 (3.6)	0.399
Symptomatic – N (%)	28 (57.1)	21 (51.2)	6 (75.0)	0.220	NA	NA	NA
pRTA – N (%)	1 (2.0)	NA	NA	NA	0	1 (3.6)	0.384
dRTA – N (%)	8 (16.3)	NA	NA	NA	2 (9.5)	6 (21.4)	0.269
Diagnosis of ATTR (years)	3 (1–9)	3 (1–10)	2 (2–9)	0.822	2 (1–7)	5 (1–14)	0.177
Hepatic transplant – N (%)	9 (18.4)	8 (19.5)	1 (12.5)	0.643	0 (0)	9 (32.1)	**0.005**
Tafamidis use – N (%)	6 (12.2)	4 (9.8)	2 (25)	0.236	0 (0)	6 (21.4)	**0.025**
Arterial blood pressure							
SBP – mmHg	115 (110–130)	115 (110–127.5)	120 (110–130)	0.968	120 (110–130)	110 (109–120)	0.144
DBP – mmHg	70 (60–77.5)	70 (60–80)	70 (60–70)	0.234	70 (60–75)	70 (60–79)	0.627
Comorbid conditions – N (%)							
Hypertension	11 (22.4)	10 (24.4)	1 (12.5)	0.465	4 (19.0)	7 (25.0)	0.622
Diabetes	6 (12.2)	6 (14.6)	0 (0)	0.253	1 (4.8)	5 (17.9)	0.171
Dyslipidemia	9 (18.4)	8 (19.5)	1 (12.5)	0.643	1 (4.8)	7 (25.0)	0.061
PACE	2 (4.1)	1 (2.4)	1 (12.5)	0.186	0 (0)	2 (7.1)	0.217
Nephrolithiasis	6 (12.2)	5 (12.2)	1 (12.5)	0.981	1 (4.8)	5 (17.9)	0.171
Smoking	7 (14.3)	6 (14.6)	1 (12.5)	0.878	1 (4.8)	6 (21.4)	0.104
Urinary incontinence	5 (10.2)	4 (9.8)	1 (12.5)	0.820	0 (0)	5 (17.9)	**0.043**
Death – N (%)	3 (6.1)	2 (4.8)	1 (12.5)	0.406	0 (0)	3 (10.7)	0.126

Abbreviations – BMI: body mass index, pRTA: proximal renal tubular acidosis, dRTA: distal renal tubular acidosis, SBP: systolic blood pressure, DBP: diastolic blood pressure, PACE: pacemaker. Note – Data are reported as mean ± SD, median [interquartile range], or proportions as appropriate.

**Table 2:** Serum laboratory tests

	TOTAL (n = 49)	No dRTA (n = 41)	dRTA (n = 8)	“p” value	Asymptomatic (n = 21)	Symptomatic (n = 28)	“p” value
eGFR CKD-EPI (ml/min/1.73m^2^)	85.5 ± 20.5	86.4 ± 21.4	89.9 ± 22.9	0.677	91.1 ± 14.6	82.9 ± 23.7	0.173
Urea (mg/dL)	29.5 (23.3–36.5)	30 (24–37)	24 (21–40)	0.358	30.0 (25.5–34.0)	27.0 (23.0–40.0)	0.708
Creatinine (mg/dL)	0.9 (0.7–1.1)	0.9 (0.7–1.1)	0.9 (0.8–1.1)	0.864	0.9 (0.8–0.9)	0.9 (0.7–1.1)	0.391
Hemoglobin (g/dL)	13.5 ± 1.5	13.6 ±1.5	13.4 ± 1.7	0.857	13.2 ± 1.2	13.8 ± 1.7	0.148
HbA1c (%)	5.5 (5.1–6.0)	5.5 (5.3–6.1)	5.1 (5.0–5.4)	**0.031**	5.4 (5.2–5.7)	5.5 (5.1–6.2)	0.292
Serum Na (mmol/L)	140 (139–142)	140 (138–142)	140 (138–144)	0.909	140 (138–142)	140 (140–143)	0.200
Serum K (mmol/L)	4.4 ± 0.4	4.4 ± 0.4	4.5 ± 0.2	0.485	4.3 ± 0.3	4.5 ± 0.4	**0.025**
Serum Ca (mg/dL)	9.5 ± 0.5	9.5 ± 0.4	9.6 ± 0.4	0.710	9.3 ± 0.5	9.6 ± 0.5	0.076
Serum P (mg/dL)	3.5 (3.3–4.0)	3.7 (3.4–4.1)	3.5 (3.1–3.7)	0.529	3.7 (3.3–4.1)	3.5 (3.3–3.8)	0.440
Serum Cl (mmol/L)	103 (100–104)	103 (100–104)	102 (99–104)	0.977	103 (100–104)	103 (101–104)	0.883
Serum Mg (mg/dL)	2.1 (1.9–2.3)	2.1 (1.9–2.3)	2 (1.9–2.3)	0.988	2.1 (2.0–2.2)	2.0 (1.9–2.3)	0.234
Serum 25(OH)D3 (ng/mL)	27.7 ± 6.8	28.0 ± 6.9	27.0 ± 4.0	0.777	29.0 ± 7.7	26.7 ± 5.9	0.254
Serum Albumin (g/dL)	4.3 (4.0–4.6)	4.3 (4.0–4.7)	4.3 (4.2–4.5)	0.775	4.4 (4.0–4.5)	4.3 (4.2–4.7)	0.484
Serum Uric acid (mg/dL)	4.6 (3.6–5.9)	4.4 (3.6–5.9)	5.0 (3.8–6.2)	0.389	4.3 (3.6–5.4)	5.0 (3.6–5.1)	0.205
AST (U/L)	19 (15–33)	20 (15–32)	13 (11–18)	**0.039**	16 (14.5–20.5)	24 (16–26)	0.053
ALT (U/L)	20 (16–29)	20 (15–28)	18 (12–24)	0.422	19 (13–26.5)	22 (18–34)	**0.037**
ALP (U/L)	70 (55–107)	76 (60–118)	58 (50–67)	0.085	66 (47.5–74.5)	97 (60–138)	**0.011**
Serum HCO_3_ (mmol/L)	25.5 ± 2.7	25.7 ± 3.0	24.5 ± 1.6	0.665	25.6 ± 3.0	25.4 ± 2.5	0.971
Serum anion gap (mmol/L)	12.3 ± 4.6	11.9 ± 4.6	13.0 ± 4.1	0.627	11.6 ± 5.0	12.7 ± 4.3	0.429

Abbreviations – NA: not applicable; HbA1C: glycated hemoglobin; 25(OH)D: 25hydroxy vitamin D; AST: aspartate aminotransferase; ALT: alanine aminotransferase; ALP: alkaline phosphatase; HCO_3_: bicarbonate; AG: anion gap. Note – Data are reported as mean ± SD, median [interquartile range], or proportions as appropriate.

**Table 3:** Urine laboratory tests

	TOTAL (n = 49)	No dRTA (n = 41)	dRTA (n = 8)	“p” value	Asymptomatic (n = 21)	Symptomatic (n = 28)	“p” value
Urine output (mL/24h)	1687 ± 677	1748 ± 679	1474 ± 658	0.371	1531.1 ± 594.7	1521.2 ± 702.3	0.310
Measured GFR (mL/min/1.73m^2^)	90.5 ± 30.5	93.9 ± 30.6	74.2 ± 29.1	0.125	97.6 ± 22.1	86.1 ± 32.9	0.069
Proteinuria (mg/24h)	141.3 ± 104.2	143.2 ± 106.8	142.4 ± 100.6	0.975	116.8 ± 58.4	151.8 ± 122.5	0.350
Urine citrate (mg/24h)	396 (271–626)	405 (297–629)	230 (136–566)	0.159	461 (308–691)	329 (257–542)	0.075
Urine HCO_3_ (mmol/L)	0.7 ± 2.1	0.5 ± 1.6	2.0 ± 4.0	0.075	1.1 ± 3.9	0.3 ± 0.7	0.165
Urine anion gap (mmol/L)	40.0 ± 47.1	35.9 ± 44.9	62.7 ± 58.5	0.167	45 ± 46.7	29.7 ± 38.3	0.645
FE Na (%)	0.8 (0.6–1.2)	0.7 (0.5–1.1)	1.1 (0.9–1.7)	**0.041**	0.9 (0,6–1.2)	0.9 (0.5–1.0)	0.596
FE K (%)	7.1 (4.7–9.8)	6.9 (4.2–9.4)	7.2 (7–13)	0.286	7.3 (4.1–12.7)	6.9 (4.5–8.7)	0.748
FE Ca (%)	1.7 (1.2–2.4)	1.8 (1.3–2.3)	1.5 (0.9–2.9)	0.546	1.9 (1.6–3.2)	1.6 (1.0–2.0)	0.135
PRR (%)	87.6 (84.3–91.5)	87.6 (83.9–91.5)	88.9 (87.0–90.6)	0.591	87.4 (85.7–91.5)	88.4 (84–91.7)	0.818
FE Mg (%)	3.3 (2.6–5.2)	3.1 (2.5–5.2)	4.6 (3.4–5.7)	0.229	3.8 (2.6–5.7)	2.8 (2.5–4.7)	0.352
FE Uric Acid (%)	6.7 (5.0–8.8)	6.6 (4.9–8.7)	7.1 (5.8–10.4)	0.546	7.6 (6.2–8.8)	5.7 (4.7–8.3)	0.389
Urine pH (dipstick)	5.0 (5.0–6.0)	5.0 (5.0–5.5)	6.5 (6.0–6.5)	**<0.001**	5.0 (5.0–5.5)	5.0 (5.0–6.0)	0.199
Urine pH 0h (potenciometry)	5.3 (5.1–5.8)	5.2 (5.0–5.5)	6.2 (5.8–6.5)	**<0.001**	5.2 (5.1–5.4)	5.5 (5.0–5.9)	0.374
Urine NH_4_ 0h (mEq/min/1.73m^2^)	30.1 (20.8–41.4)	29.1 (20–36.6)	46.4 (30.5–53.6)	**0.016**	29.5 (23.4–38.6)	30.5 (19.9–44.9)	0.944
Urine TA 0h (mEq/min/1.73m^2^)	21.3 (13.6–26.8)	21.6 (13.3–26.4)	19.0 (15.7–23.6)	0.968	22.4 (14.2–33.4)	18.9 (13.3–23.5)	0.164
PNA (gPtn/kg/24h)	1.3 (1.1–1.8)	1.3 (1.1–1.8)	1.2 (1.1–2.2)	0.203	1.4 (1.0–1.6)	1.3 (1.1–2.4)	0.455
NaCl intake (g/24h)	6.8 (5.7–11.8)	6.8 (5.8–11.8)	7.2 (3.4–8.6)	0.529	6.7 (5.7–10.4)	6.9 (5.4–11.8)	0.800

Abbreviations – FE: fractional excretion; PRR: phosphate reabsorption rate; PNA: protein nitrogen appearance; pH 0 h = pH before urinary acidification protocol; NH_4_^+^ 0h = urine ammonium before urinary acidification protocol; TA 0 h = titratable acidity before urinary acidification protocol. Note – Data are reported as mean ± SD, median [interquartile range], or proportions as appropriate.

**Table 4:** individual data of subjects with drta (n = 8)

	Subject #1	Subject #2	Subject #3	Subject #4	Subject #5	Subject #6	Subject #7	Subject #8
Symptomatic	Yes	Yes	Yes	Yes	No	No	Yes	Yes
Liver transplant	No	No	Yes	No	No	No	No	No
Use of Tafamidis	Yes	No	No	No	No	No	Yes	No
Serum HCO_3_ (mmol/L)	24.9	26.7	23.0	26.0	23.1	22.2	24.9	30.0
eGFR CKD-EPI (mL/min/1.73m^2^)	120	82	60	100	120	79	94	64
Proteinuria (mg/24h)	104	330	100	150	210	75	28	NA
Urine citrate (mg/24h)	196	155	77	264	521	699	NA	NA
Urine pH (dipstick)	6.50	6.00	6.50	6.50	8.00	6.00	6.00	6.00
Urine pH 0h (potenciometry)	6.24	5.83	6.50	6.20	7.10	5.77	5.55	5.75
Urine pH 1h (potenciometry)	6.47	5.69	6.44	6.89	6.29	6.17	5.79	5.72
Urine pH 2h (potenciometry)	5.36	6.12	6.73	6.39	6.22	6.51	6.30	6.11
Urine pH 3h (potenciometry)	6.10	5.96	6.54	5.60	6.64	5.86	5.69	5.80
Urine pH 4h (potenciometry)	6.24	6.11	6.12	6.40	5.90	6.42	6.14	6.56
Urine NH_4_ 0h (mEq/min/1.73m^2^)	169	46.4	30.5	**53.6**	45.9	19.5	48	40.3
Urine NH_4_ 4h (mEq/min/1.73m^2^)	9.0	45.1	78.4	**65.8**	11.5	62.7	11.5	173.6
Urine TA 0h (mEq/min/1.73m^2^)	68.3	23.6	15.7	23.5	11.6	19	17.1	14.4
Urine TA 4h (mEq/min/1.73m^2^)	15.2	18.8	36.2	21.6	16.6	27.4	16.6	8.6

Abbreviations – NA: not applicable; pH 0h: pH before urinary acidification protocol; NH_4_^+^ 0h = urine ammonium before urinary acidification protocol; NH_4_^+^ 4h = urine ammonium after urinary acidification protocol, TA 0h: titratable acidity before urinary acidification protocol, TA 4h: titratable acidity after urinary acidification protocol.

The study population was composed of young individuals, mostly female, Caucasian, with laboratory results within the normal range. 87.7% (n = 44) of the patients had preserved renal function, with creatinine of 0.9 (0.7–1.1) mg/dL and eGFR > 60 mL/min/1.73m^2^. None had systemic metabolic acidosis. Most had the Val50Met mutation (94%) and had been diagnosed for a mean of 3 years. There was a low prevalence of pre-existing dise ases of any kind. Approximately half of the patients were symptomatic. Although 34.7% (n = 17) had proteinuria ≥150 mg on 24-hour urine analysis, the mean proteinuria remained within the normal range. Only 8.1% (n = 4) had albuminuria above 30 mg/day.

Of the 49 patients evaluated, 9 had some degree of renal acidification dysfunction: 8 patients had incomplete dRTA (16.3%) and 1 patient had pRTA (2%). In the dRTA subgroup, 2 patients were asymptomatic.

There was no significant difference between the subgroups with and without dRTA from an epidemiologic point of view. However, when comparing symptomatic and asymptomatic subgroups, individuals in the asymptomatic subgroup were younger. The other three parameters with a significant difference were: use of tafamidis, previous liver transplant, and urinary incontinence, which were present only in the symptomatic group.

In the laboratory results, there was a significant difference between patients with and without dRTA only in HbA1c and AST, but this did not seem to have a clinical impact. Between the symptomatic and asymptomatic subgroups, there was a difference in potassium levels, also without apparent clinical impact, and in ALT and ALP levels, which were higher in the symptomatic group, probably due to liver transplant patients. The average proteinuria of the total number of patients was within the normal range.

In urine analysis, we found higher FENa in the dRTA group. There was no significant difference in urinary citrate between the groups with and without dRTA.

**Figure 1:**
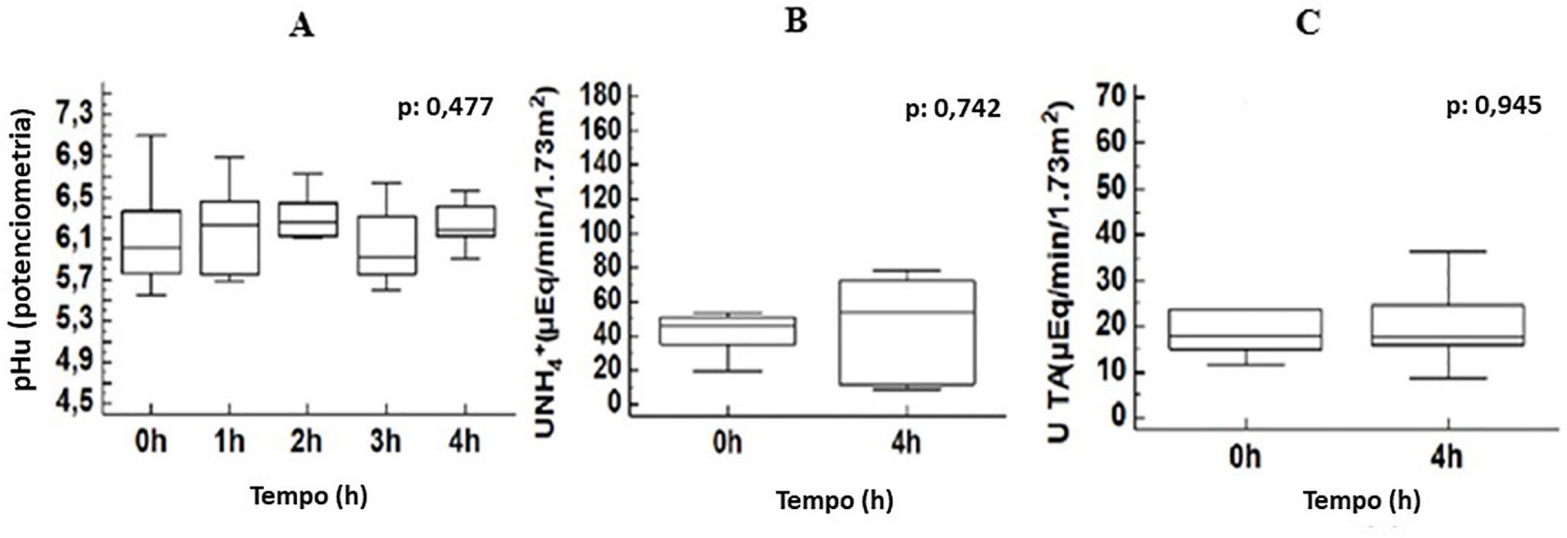
(A) Urine pH curve by potentiometry in the dRTA group. (B) Urine ammonium pre (0h) and post (4h) urinary acidification test in dRTA group. (C) Urine titratable acidity pre (0h) and post (4h) urinary acidification test in dRTA group.

Baseline urine pH was significantly higher in the dRTA group for both dipstick and potentiometric measurements, and baseline urine ammonia was also higher in the dRTA group. There was no difference in baseline titratable acidity between these two subgroups. In Figure 1, we present the urine pH curve of patients with dRTA, showing no significant decrease (UpH <5.3) during the acidification challenge. As also shown in Figure 1, there was no increase in either UTA or ${\text{UNH}}_{4}^{+}$ after the acid challenge in this group of patients with dRTA.

**Figure 2:**
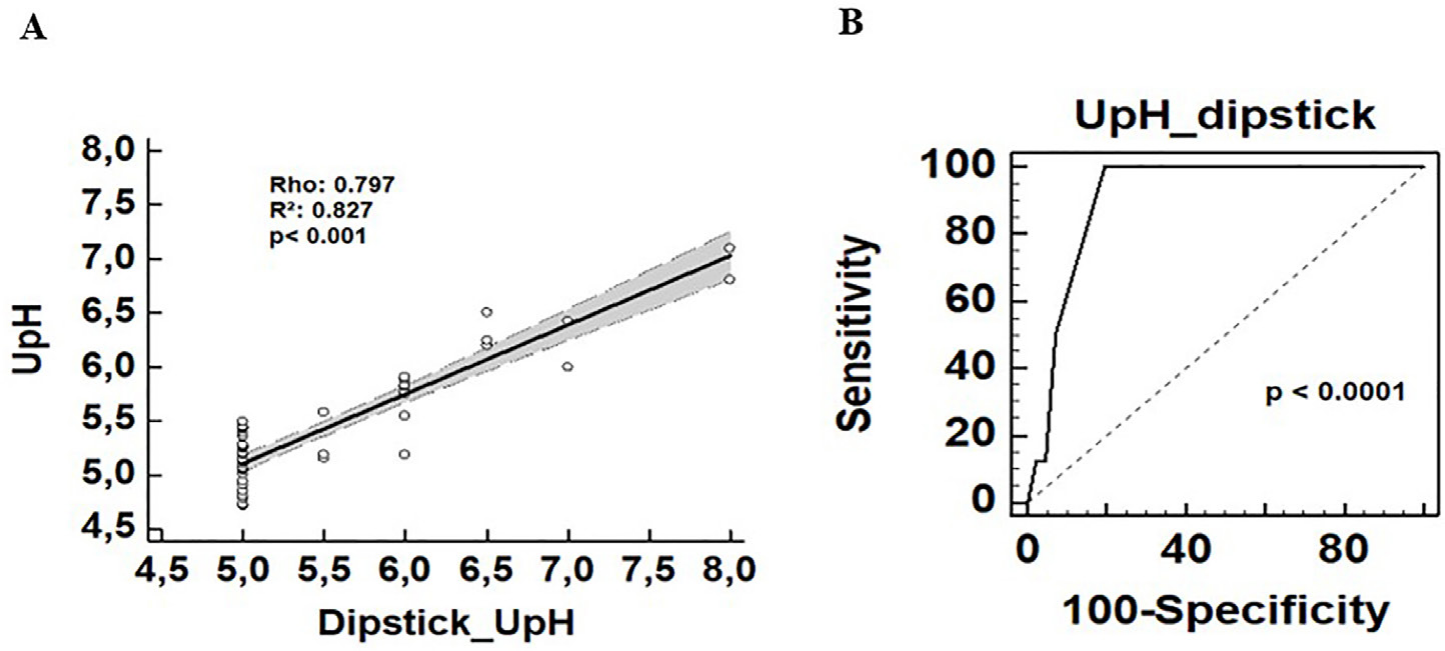
(A) Linear correlation curve between UpH (urine pH) by dipstick versus potentiometry. (B) ROC curve: Urine pH by dipstick < 5.5 to rule out diagnostic of dRTA.

Furthermore, we obtained a highly significant correlation between dipstick UpH and UpH potentiometry (Spearman’s Rho 0.797, p<0.001), as shown by the regression line in Figure 2. Based on the ROC curve, the best cut-off point in the dipstick to exclude dRTA was UpH < 5.5, with 100% sensitivity and 81% specificity.

## Discussion

This cross-sectional study included outpatients with ATTRv followed by a multidisciplinary team at a reference center for ATTRv. Patients with ATTRv usually have preserved glomerular renal function, which was one of our inclusion criteria. We found a prevalence of 18.3% of RTA in this population, mostly dRTA, which is usually a subclinical condition and may precede the decline in GFR.

Considering amyloidosis in general, there are several possibilities for damage in each of the renal compartments. Among tubular disorders, the most common is type 2 RTA as a manifestation of Fanconi syndrome in AL amyloidosis, which is due to impaired proteolysis of light chains in the proximal tubule, impairing its absorptive function^21^. There are a few reports of type 4 RTA, mainly in AA amyloidosis^22^. However, considering only ATTRv, much of the literature on renal involvement is based on small series describing mainly proteinuria and GFR impairment. None of these studies report data on tubular function.

According to Lobato and Rocha^6^, the occurrence and onset of nephropathy in ATTRv varies according to the mutation. In our study, we focused on the V50M mutation, which is the most common mutation in Brazil. In this mutation, renal failure usually occurs 5 years after microalbuminuria, even earlier in women^5^. A crucial issue in ATTRv nephropathy is the recognition of the presence and distribution of renal amyloid deposition^6^. A few studies have systematically evaluated renal histopathology in ATTRv Val50Met and showed that, in addition to the glomerular compartment, the medulla, basement membrane of distal tubules, loops of Henle, and interstitium are typically filled with amyloid deposits, even in the early stages of the disease^7^. Glomerular deposits are discrete and often cause albuminuria, which is well described in the literature. Patients with albuminuria had more extensive amyloid involvement than those without clinical renal disease^8^. Renal dysfunction and the degree of proteinuria correlate with heavy amyloid deposition in glomeruli, arterioles, and middle vessels, but not with deposition in medullary tissues^6^. On the other hand, amyloid deposition is present in the tubulointerstitial compartment even in patients with normal levels of albuminuria^7^.

It is therefore necessary to assess whether tubulointerstitial function is also impaired, since the presence of deposits in this region has been demonstrated. This can be a tool for early diagnosis of the renal manifestation of ATTRv, since it is a subclinical alteration. The search for early diagnostic tools has become relevant in recent years due to new treatment options that have prolonged patient survival. In addition, most of the currently available treatments only stabilize the disease, so they can only be initiated in patients who already have some clinical manifestations^2^. Therefore, early diagnosis is crucial for early treatment.

Our population was mainly female and Caucasian, given that this is a disease typically prevalent in Portuguese people and their descendants. The mean BMI was normal in the general population and in each subgroup, although the symptomatic subgroup had a lower BMI, although without statistical significance. This may be due to the typical dysautonomia with significant delay in gastric emptying and chronic diarrhea. Liver transplant patients or patients receiving Tafamidis were only included in the symptomatic subgroup, as these are treatments used only in clinically manifest disease^1^. To assess the possible effect of calcineurin inhibitors or Tafamidis on tubular function, we compared clinical and laboratory parameters of patients taking or not taking these medications, with no significant difference (data not shown).

Due to Portuguese colonization in Brazil, the most common mutation in our cohort was Val50Met. The prevalence of pre-existing, potentially kidney-damaging diseases was low, suggesting that the results shown are due to ATTRv nephropathy. Proteinuria and eGFR estimated by the CKD-EPI formula showed mostly preserved glomerular renal function in our population, supporting that our findings may precede the more classically described renal changes.

Despite the normal GFR of all included patients, we observed a high prevalence of low urinary acidification capacity, mainly dRTA (16.3%). It is interesting to note that none of the patients diagnosed with dRTA had systemic metabolic acidosis, the so-called incomplete form of dRTA. The absence of acidemia may justify an oligosymptomatic or even asymptomatic presentation.

Chronic metabolic acidosis may have adverse effects on nutritional status and bone metabolism. Although we did not find systemic metabolic acidosis, it is important to emphasize that even the incomplete form of dRTA (without systemic acidosis) can lead to bone loss and an increased risk of nephrolithiasis due to calcium phosphate stones^23^. Both patients with incomplete and complete renal tubular acidosis experience bone demineralization secondary to bone buffering. Administration of potassium bicarbonate to menopausal women has been shown to result in a positive calcium balance and improved bone densitometry, which is an indirect indicator of osteomineral damage caused by chronic metabolic acidosis^24^. Furthermore, decreased ${\text{UNH}}_{4}^{+}$ excretion is associated with a higher risk of end-stage CKD and a rapid decline in GFR, suggesting that the inability to excrete the daily acid load is detrimental to renal prognosis^25^. Therefore, it is important to diagnose dRTA early to allow for early treatment and avoid the detrimental consequences of this disorder.

Tables 1, 2, and 3 show the comparison between symptomatic versus asymptomatic patients and patients with dRTA versus without dRTA from the epidemiologic, laboratory, and urinalysis point of view. The asymptomatic group had younger individuals, probably because of family screening performed on relatives of confirmed cases. Although hypokalemia is classically described in dRTA, we did not observe this disorder in our patients, and there was no significant difference in serum potassium levels between the groups with and without dRTA.

UTA is the amount of H^+^ secreted by the cells of the distal tubule and collecting duct, bound to the neutral salts filtered by the glomerulus, giving rise to the acidic salts in the urine TA is not the only available pathway for acid excretion^26,27^. Another mechanism is the excretion of ${\text{UNH}}_{4}^{+}$ . Both allow acid excretion without excessive urinary acidity, with only about 1% of the H^+^ load being excreted in free form^26,27^. There was no significant increase in ${\text{UNH}}_{4}^{+}$ or UTA in response to the urinary acidification protocol in the subgroup with dRTA.

Although there was no statistically significant difference between the median urinary citrate levels of the subgroups with and without dRTA, only the subgroup with dRTA showed a level considered hypocitraturic (<340 mg/24h). Furthermore, it was 43.2% lower compared to the subgroup without dRTA. The lack of statistical significance may be due to the small number of our sample, which caused a high dispersion in this analysis. Hypocitraturia is classically associated with dRTA and helps to confirm the diagnosis. It occurs due to avid citrate recovery by proximal tubular cells in the setting of systemic acidosis^13^. Shavit and colleagues, in 2016 revision of Walsh’s 2007 protocol, even suggest that the combination of an abnormal furosemide and fludrocortisone test associated with hypocitraturia and alkaline urine increases the diagnostic probability of dRTA and may avoid ammonium chloride testing^17^. In addition, there was a tendency for FENa to be slightly higher in individuals with dRTA, which may exacerbate hypotension in patients who already have dysautonomia. Although polyuria has been described as a possible symptom of distal renal tubular acidosis, none of our patients with distal RTA presented this clinical manifestation. Although there were 9 liver transplant patients, only one had dRTA. It is known that calcineurin inhibitors may affect tubular function, but in our sample this was irrelevant.

An interesting finding was the significant correlation between xurinary pH measured by dipstick and potentiometer. The dipstick is useful for screening as potentiometry is not widely available in clinical practice. Therefore, it expands access to a reliable measurement of UpH in this setting. In addition, we found that the measurement of UpH < 5.5 using a single reagent strip had a sensitivity of 100% and a specificity of 93.9% to rule out the diagnosis of dRTA.

A limitation of this study was that we did not use ammonium chloride overload^11,28,29^ after the furosemide and fludrocortisone protocol to avoid the potential side effects of induced metabolic acidosis, such as nausea, vomiting, hemodynamic instability, etc. Our patients might have already had dysautonomia with a tendency to hypotension, and some of them had a history of liver transplantation, increasing the risk of side effects from the use of ammonium chloride. Another problem was that our population was mostly composed of only one type of mutation or pathogenic genetic variant (Val50Met). Therefore, the results cannot be extrapolated to other genetic variants.

To our knowledge, this is the first study to demonstrate renal tubular dysfunction in ATTRv patients as assessed by urinary acidification capacity. We found a high prevalence of subclinical ATTRv and suggest how to diagnose this early renal manifestation of ATTRv. Diagnosis of subclinical ATTRv in patients with Val50Met mutation may allow specific treatment not only to avoid the complications of dRTA, but also to introduce new agents that stabilize or reduce the production of the mutant TRR. These patients diagnosed with dRTA will be followed up in the coming years. Further studies are needed to evaluate the impact of drug treatment on the progression of nephropathies in patients with ATTRv.
