# Supplementary material for: Renal tubular acidosis in hereditary transthyretin amyloidosis (ATTRv)
Source: J Bras Nefrol. 2024 Jul 22;46(4):e20240016. doi: 10.1590/2175-8239-JBN-2024-0016en (PMC11305565; doi:10.1590/2175-8239-JBN-2024-0016en)
Supplement: Translation

# Translation: Acidose tubular renal na amiloidose hereditária por transtirretina (ATTRv)

**DOI:** 10.1590/2175-8239-JBN-2024-0016pt

**Authors:** Priscilla Cardim Fernandes, Moises Dias da Silva, Marcia Waddington-Cruz, Carlos Perez Gomes

**Affiliations:** 1 Universidade Federal do Rio de Janeiro, Hospital Universitário Clementino Fraga Filho, Centro de Estudos em Paramiloidose Antônio Rodrigues de Mello, Rio de Janeiro, RJ, Brasil.; 2 Universidade Federal do Rio de Janeiro, Hospital Universitário Clementino Fraga Filho, Divisão de Nefrologia, Rio de Janeiro, RJ, Brasil.; 3 Universidade Federal do Estado do Rio de Janeiro, Escola de Medicina e Cirurgia, Rio de Janeiro, RJ, Brasil.

**Keywords:** Neuropatias amiloides familiares, miloidose Familiar, Acidose Tubular Renal, Pré-Albumina, Urinálise

## Abstract

**Introdução:**

A amiloidose hereditária por transtirretina (ATTRv) é uma doença sistêmica autossômica dominante grave. Afeta os sistemas nervoso periférico e autônomo, coração, rins e olhos. A deposição de amiloide foi demonstrada nos compartimentos glomerular e tubulointersticial do rim. Portanto, distúrbios de acidificação urinária, como acidose tubular renal (ATR), podem ser manifestações precoces de envolvimento renal nessa população.

**Objetivo:**

Avaliar a prevalência de ATR em indivíduos com ATTRv.

**Métodos:**

Incluímos indivíduos sintomáticos e assintomáticos com mutação na TTR, maiores de 18 anos, TFG >45 mL/min/1,73m^2^, sem acidose metabólica sistêmica. Realizou-se protocolo de acidificação urinária com furosemida e fludrocortisona após 12 horas de privação hídrica (teste de restrição hídrica - TRH) e medições de amônia urinária ( ${\text{uNH}}_{4}^{+}$ ) e acidez titulável (uTA) na urina. ATR proximal (ATRp) foi diagnosticada quando FEHCO_3_>10%. ATR distal (ATRd) de forma incompleta foi diagnosticada se pHu>5,3.

**Resultados:**

Selecionamos 49 indivíduos com idade média de 40 (35,5–56,5) anos, 63% mulheres, 84% caucasianos e TFG média de 85,5 ± 20,5 mL/min/1,73m^2^. 94% apresentaram a variante genética Val50Met; 57% eram sintomáticos. A prevalência de ATRp foi 2% e a de ATRd foi 16,3%. No subgrupo com ATRd, não houve aumento significativo na excreção de ${\text{uNH}}_{4}^{+}$ e uTA. Observamos uma boa correlação entre pHU por potenciometria e pHU por fita reagente. Um pHU<5,5 na fita reagente apresentou 100% de sensibilidade e valor preditivo negativo para excluir a ATRd.

**ConclusÃO:**

Uma alta prevalência de ATR foi encontrada em indivíduos com mutações na TTR. O pHU por fita reagente após TRH teve boa precisão para triagem de ATRd. São necessários mais estudos para avaliar o impacto do diagnóstico e tratamento precoces da ATR nessa população.

## Introdução

A amiloidose hereditária por transtirretina (ATTRv) é uma doença autossômica dominante com penetrância variável e é a forma mais comum de amiloidose hereditária. Atualmente, existem cerca de 120 mutações diferentes que produzem TTR mutante, das quais apenas cerca de dez tipos são não patogênicos, com Val50Met sendo a mutação mais comum^1^.

A ATTRv é uma doença multissistêmica, progressiva e incapacitante. A apresentação clínica é caracterizada pelo envolvimento do sistema nervoso periférico, coração, sistema digestivo, rins e olhos. A apresentação clínica é variável, podendo variar desde o envolvimento neurológico exclusivo até casos esporádicos com manifestações estritamente cardiológicas^2^. O curso clássico da ATTRv consiste em polineuropatia sensório-motora e autonômica, disfunção gastrointestinal, bloqueio de condução cardíaca, cardiomiopatia infiltrativa (cardiomiopatia amiloidótica familiar), nefropatia e, menos comumente, deposição ocular que leva a opacidades vítreas ou glaucoma^3^. A idade mediana de início ocorre na quarta e quinta décadas. Sem tratamento, o tempo mediano desde o início dos sintomas até o óbito é de aproximadamente 10 a 15 anos^4^.

Inicialmente considerada uma doença benigna em termos de função renal, reconheceu-se posteriormente que a progressão para doença renal em estágio terminal ocorre como uma história natural em até um terço dos pacientes portadores da mutação Val50Met^5^. A nefropatia é mais comum em mulheres, em pacientes com neuropatia de início tardio, em casos com baixa penetrância familiar e em pacientes com cardiomiopatia preexistente^6^. Uma questão crítica na nefropatia por ATTRv é o reconhecimento da presença e distribuição da deposição de amiloide renal. Os glomérulos, a medula, a membrana basal dos túbulos distais, as alças de Henle e o interstício são tipicamente preenchidos por depósitos amiloides, mesmo nos estágios iniciais da doença. A disfunção renal e a proteinúria são proporcionais ao grau de deposição de amiloide nos glomérulos, arteríolas e vasos médios. Entretanto, eles não se correlacionam com depósitos na medula renal^7^. Atualmente, o diagnóstico de nefropatia amiloide pode ser inferido a partir da presença de albuminúria em pacientes com neuropatia documentada, embora o padrão ouro para o diagnóstico definitivo seja a histopatologia renal. Contudo, a albuminúria não é um marcador específico de dano tubulointersticial e pode estar ausente em cerca de 10% dos pacientes que progridem para doença renal em estágio terminal^8,9^. Até o momento, não foram descritas tubulopatias na ATTRv.

A acidose tubular renal (ATR) é uma disfunção tubular e consiste em uma incapacidade de reabsorver o HCO_3_ filtrado e/ou excretar uma carga ácida pelos rins, comprometendo a manutenção do equilíbrio ácido-base^10^. Distingue-se da acidose urêmica pela presença de uma taxa de filtração glomerular preservada ou ligeiramente reduzida e por ser uma acidose hiperclorêmica com ânion gap normal^11^. Existem cinco tipos de ATR: O tipo 2 ou ATR proximal (ATRp) é caracterizado pela reabsorção ou regeneração prejudicada de HCO_3_ no túbulo proximal, o que reduz o ponto de ajuste da reabsorção de HCO_3_ e aumenta a fração de excreção de HCO_3_ (>10%) mesmo com um nível sérico normal, apresentando normocalemia/hipocalemia^12^. O tipo 1 clássico ou ATR distal (ATRd) é caracterizado pela acidificação distal da urina prejudicada, sem diminuição do pH urinário na presença de acidemia ou mesmo após sobrecarga ácida^13^, e também é caracterizado por normocalemia ou hipocalemia. Por outro lado, a ATR distal ou tipo 1 voltagem dependente é caracterizada por hipercalemia. A ATR tipo 3 é rara e apresenta características das formas distal e proximal. A ATR tipo 4 ou hipercalêmica é causada por deficiência de aldosterona ou resistência tubular a esse hormônio^14^.

Portanto, é pertinente avaliar se a população afetada apresenta um distúrbio de acidificação urinária, uma vez que essa disfunção tubular é possível devido a depósitos medulares e pode ser uma ferramenta valiosa no diagnóstico precoce de envolvimento renal na ATTRv. O objetivo deste estudo foi analisar a prevalência de ATR em pacientes com ATTRv e comparar diferentes subgrupos de pacientes de acordo com o diagnóstico de ATR e a presença de sintomas extrarrenais.

## Métodos

Um estudo transversal foi desenhado para avaliar a prevalência de distúrbios subclínicos de acidificação urinária em indivíduos com mutação na TTR acompanhados no Centro de Estudos de Paramiloidose Antonio Rodrigues de Mello (CEPARM), localizado no Hospital Universitário Clementino Fraga Filho (HUCFF) da Universidade Federal do Rio de Janeiro – UFRJ, Brasil. Este estudo foi aprovado pelo comitê de ética local do HUCFF-UFRJ (Projeto número 47772021.5.0000.5257) e foi conduzido de acordo com a Declaração de Helsinque.

Foram incluídos indivíduos assintomáticos e sintomáticos maiores de 18 anos com TFGe > 45 mL/min/1,73m^2^ estimada pela equação CKD-EPI (*Chronic Kidney Disease Epidemiology Collaboration*) e controle miccional preservado. Excluímos pacientes com infecção ativa do trato urinário, infecção por HIV (vírus da imunodeficiência humana), HBV (vírus da hepatite B) ou HCV (vírus da hepatite C), doença pulmonar crônica, diarreia aguda, doenças autoimunes, gestantes e pacientes com contraindicações ao uso de furosemida ou fludrocortisona. Os pacientes foram avaliados entre março de 2019 e fevereiro de 2020.

Consideramos pacientes sintomáticos aqueles com sintomas relacionados à polineuropatia sensório-motora, disautonomia, distúrbios de condução cardíaca, arritmias ou disfunção sistólica ou diastólica. Os pacientes assintomáticos foram aqueles com teste genético positivo para a mutação, mas sem nenhum dos sintomas ou sinais acima^1,2,3^.

Todos os pacientes fizeram sua última refeição às 20h do dia anterior e permaneceram em jejum completo de líquidos e sólidos até as 8h da manhã para garantir a concentração máxima de urina por 12 horas (teste de restrição hídrica - TRH). Às 8h00, foi coletada uma amostra da primeira urina (amostra basal) para as seguintes medições: pH urinário (pHU), pCO_2_ e bicarbonato urinário (uHCO_3_) por gasometria urinária (potenciometria), amônia ( ${\text{uNH}}_{4}^{+}$ ) por espectrofotometria, ácido titulável (uTA) por NaOH em bureta e urinálise por fita reagente. Amostras de urina fresca foram coletadas e analisadas imediatamente para evitar a perda de CO_2_. Uma amostra de sangue venoso também foi coletada neste momento inicial para análise de gases sanguíneos e exames laboratoriais subsequentes: hemograma completo, glicose, hemoglobina glicada (HbA1c), sódio, potássio, cálcio, magnésio, fosfato, cloreto, ácido úrico, albumina, ureia, creatinina, aspartato (AST) e alanina (ALT) aminotransferases, fosfatase alcalina (FA) (45-129 U/L) e 25-hidroxi-vitamina D (25(OH)D3) (>30 ng/mL). Para os pacientes que não atingiram pH urinário basal < 5,5 por potenciometria após o TRH, utilizamos um protocolo modificado descrito por Wash et al. para avaliar a capacidade reduzida de acidificação urinária do néfron distal^11^. Esse teste de estresse de acidificação consiste na administração de 40 mg de furosemida e 0,1 mg de fludrocortisona, seguido de coleta de urina a cada hora durante 4 horas para medição do pH (0, 1, 2, 3 e 4 h). Na última amostra de urina (4 h), ${\text{uNH}}_{4}^{+}$ e uTA também foram medidos para comparação com a amostra basal. A ATRp foi definida como FEHCO_3_ > 10%^15^. A ATRd incompleta foi definida por pHU > 5,3 em todas as medições e nenhum aumento em ${\text{uNH}}_{4}^{+}$ (26-28 mEq/min/1,73m^2^) ou uTA (22-47 mEq/min/1,73m^2^)^16^ após o teste de acidificação urinária^11,17^.

Na mesma semana, os pacientes realizaram uma coleta de urina de 24 horas para medição de proteinúria (<150 mg/24 h), albuminúria (<30 mg/24 h), creatinina, sódio, potássio, cálcio, magnésio, fosfato, cloreto, ácido úrico e citrato (>320 mg/24 h)^18,19^. A partir das amostras de sangue e urina, foi possível calcular o ânion gap sérico [AG = Na – (Cl + HCO_3_)] (8–14 mmol/L), o ânion gap urinário [uAG = (Na + K) – Cl)] (valores negativos em mmol/L), a TFG medida (>60 mL/min/1,73m^2^), aparência de nitrogênio proteico para estimativa da ingestão de proteína [(ANP = ((ureia urinária x 0,46) + 2) × 6,25)/ peso corporal))] (0,8–1,2 g ptn/kg/24 h), estimativa da ingestão de NaCl (g/24h), e excreção fracionada (EF) de eletrólitos^11,12^. A EF de cada eletrólito é calculada pela fórmula:

$$\mathrm{EF de eletrólitos = 100 \times}\frac{\mathrm{Eletrólito urinário \times Creatinina plasmática}}{\mathrm{Eletrólito plasmático \times Creatinina urinária}}$$

Foram considerados os seguintes valores de referência para excreção fracionada: EF Na < 1%, EF K < 30%, EF Ca < 3%, taxa de reabsorção de fosfato (TRF) [(1 – EF fosfato) × 100] > 80%, EF Mg < 6%, EF ácido úrico < 10%^12,20^.

As variáveis categóricas foram expressas como números absolutos e porcentagens, e as variáveis contínuas foram expressas como média ± desvio padrão ou mediana (intervalo interquartil). O teste t não pareado ou o teste de Mann-Whitney foram utilizados para comparações entre dois grupos, e o teste de Friedman com análise post-hoc foi utilizado para comparações entre três ou mais grupos. A correlação de Spearman e a análise de regressão linear foram usadas para medir a associação entre duas variáveis. A curva ROC (*Receiver Operator Characteristic*) foi usada para avaliar a precisão do pH medido por fita reagente para o diagnóstico da ATRd. As análises estatísticas foram realizadas com o programa *IBM SPSS Statistics*, versão 24 (EUA), e o nível de significância foi fixado em 0,05 (bicaudal). O estudo foi aprovado pelo comitê de ética local e os participantes deram seu consentimento informado.

## Resultados

No momento do recrutamento, 272 pacientes estavam em tratamento no ambulatório do CEPARM. Desses, 96 foram excluídos por residirem fora do estado do Rio de Janeiro, 79 se recusaram a participar, 25 não puderam ser contatados por telefone, 9 estavam participando de um estudo duplo-cego no momento da avaliação, 8 apresentaram TFGe < 45 mL/min/1,73m^2^, 5 tinham amiloidose não relacionada à TTR e 1 estava grávida. Portanto, selecionamos 49 pacientes, dos quais 28 eram sintomáticos (57,1%) e 21 eram assintomáticos (42,9%). De todos os pacientes sintomáticos, 9 já haviam sido submetidos a transplante hepático e 6 estavam tomando tafamidis, um medicamento específico que estabiliza a proteína TTR mutante.

Os dados são apresentados para a população total, por subgrupos com base na presença ou ausência de sintomas sistêmicos (cardíacos e/ou neurológicos) e por subgrupos com e sem ATRd. As características clínicas e laboratoriais basais dos grupos sintomáticos e assintomáticos são mostradas nas Tabelas 1, 2 e 3.

**Tabela 1:** Características epidemiológicas el clínicas

	TOTAL (n = 49)	Sem ATRd (n = 41)	ATRd (n = 8)	Valor de “p”	Assintomático (n = 21)	Sintomático (n = 28)	Valor de “p”
Idade (anos)	40 (35,5–56,5)	47 (35,5–58,5)	40 (30,0–48,0)	0,550	37,0 (29,0–49,5)	49,0 (37,3–60,0)	**0,010**
Sexo feminino – N (%)	31 (63,3)	27 (65,9)	4 (50)	0,395	16 (76,2)	15 (53,6)	0,108
Cor da pele – N (%)							
Branca	41 (83,7)	34 (83,0)	7 (87,5)	0,755	20 (95,2)	21 (75,0)	0,061
Não branca	8 (16,3)	7 (17,0)	1 (12,5)	0,755	1 (4,8)	7 (25,0)	0,061
IMC (kg/m2)	24,9 ± 4,9	25,1 ± 4,6	25,1 ± 5,3	0,580	26,4 ± 4,8	24,0 ± 4,8	0,087
Mutação – N (%)							
Val50Met	46 (93,9)	38 (92,7)	8 (100)	0,435	19 (90,5)	27 (96,4)	0,399
Outras	3 (6,1)	3 (7,3)	0 (0)	0,435	2 (9,5)	1 (3,6)	0,399
Sintomático – N (%)	28 (57,1)	21 (51,2)	6 (75,0)	0,220	NA	NA	NA
ATRp – N (%)	1 (2,0)	NA	NA	NA	0	1 (3,6)	0,384
ATRd – N (%)	8 (16,3)	NA	NA	NA	2 (9,5)	6 (21,4)	0,269
Diagnóstico de ATTR (anos)	3 (1–9)	3 (1–10)	2 (2–9)	0,822	2 (1–7)	5 (1–14)	0,177
Transplante hepático – N (%)	9 (18,4)	8 (19,5)	1 (12,5)	0,643	0 (0)	9 (32,1)	**0,005**
Uso de Tafamidis – N (%)	6 (12,2)	4 (9,8)	2 (25)	0,236	0 (0)	6 (21,4)	**0,025**
Pressão arterial							
PAS – mmHg	115 (110–130)	115 (110–127,5)	120 (110–130)	0,968	120 (110–130)	110 (109–120)	0,144
PAD – mmHg	70 (60–77,5)	70 (60–80)	70 (60–70)	0,234	70 (60–75)	70 (60–79)	0,627
Condições comórbidas – N (%)							
Hipertensão	11 (22,4)	10 (24,4)	1 (12,5)	0,465	4 (19,0)	7 (25,0)	0,622
Diabetes	6 (12,2)	6 (14,6)	0 (0)	0,253	1 (4,8)	5 (17,9)	0,171
Dislipidemia	9 (18,4)	8 (19,5)	1 (12,5)	0,643	1 (4,8)	7 (25,0)	0,061
MP	2 (4,1)	1 (2,4)	1 (12,5)	0,186	0 (0)	2 (7,1)	0,217
Nefrolitíase	6 (12,2)	5 (12,2)	1 (12,5)	0,981	1 (4,8)	5 (17,9)	0,171
Tabagismo	7 (14,3)	6 (14,6)	1 (12,5)	0,878	1 (4,8)	6 (21,4)	0,104
Incontinência urinária	5 (10,2)	4 (9,8)	1 (12,5)	0,820	0 (0)	5 (17,9)	**0,043**
Óbito – N (%)	3 (6,1)	2 (4,8)	1 (12,5)	0,406	0 (0)	3 (10,7)	0,126

Abreviações – IMC: índice de massa corporal, ATRp: acidose tubular renal proximal, ATRd: acidose tubular renal distal, PAS: pressão arterial sistólica, PAD: pressão arterial diastólica, MP: marcapasso. Nota – Os dados são relatados como média ± DP, mediana [intervalo interquartil] ou proporções, conforme apropriado.

**Tabela 2:** Exames laboratoriais séricos

	TOTAL (n = 49)	Sem ATRd (n = 41)	ATRd (n = 8)	Valor de “p”	Assintomático (n = 21)	Sintomático (n = 28)	Valor de “p”
TFGe CKD-EPI (ml/min/1,73m2)	85,5 ± 20,5	86,4 ± 21,4	89,9 ± 22,9	0,677	91,1 ± 14,6	82,9 ± 23,7	0,173
Ureia (mg/dL)	29,5 (23,3–36,5)	30 (24–37)	24 (21–40)	0,358	30,0 (25,5–34,0)	27,0 (23,0–40,0)	0,708
Creatinina (mg/dL)	0,9 (0,7–1,1)	0,9 (0,7–1,1)	0,9 (0,8–1,1)	0,864	0,9 (0,8–0,9)	0,9 (0,7–1,1)	0,391
Hemoglobina (g/dL)	13,5 ± 1,5	13,6 ±1,5	13,4 ± 1,7	0,857	13,2 ± 1,2	13,8 ± 1,7	0,148
HbA1c (%)	5,5 (5,1–6,0)	5,5 (5,3–6,1)	5,1 (5,0–5,4)	**0,031**	5,4 (5,2–5,7)	5,5 (5,1–6,2)	0,292
Na sérico (mmol/L)	140 (139–142)	140 (138–142)	140 (138–144)	0,909	140 (138–142)	140 (140–143)	0,200
K sérico (mmol/L)	4,4 ± 0,4	4,4 ± 0,4	4,5 ± 0,2	0,485	4,3 ± 0,3	4,5 ± 0,4	**0,025**
Ca sérico (mg/dL)	9,5 ± 0,5	9,5 ± 0,4	9,6 ± 0,4	0,710	9,3 ± 0,5	9,6 ± 0,5	0,076
P sérico (mg/dL)	3,5 (3,3–4,0)	3,7 (3,4–4,1)	3,5 (3,1–3,7)	0,529	3,7 (3,3–4,1)	3,5 (3,3–3,8)	0,440
Cl sérico (mmol/L)	103 (100–104)	103 (100–104)	102 (99–104)	0,977	103 (100–104)	103 (101–104)	0,883
Mg sérico (mg/dL)	2,1 (1,9–2,3)	2,1 (1,9–2,3)	2 (1,9–2,3)	0,988	2,1 (2,0–2,2)	2,0 (1,9–2,3)	0,234
25(OH)D3 sérica (ng/mL)	27,7 ± 6,8	28,0 ± 6,9	27,0 ± 4,0	0,777	29,0 ± 7,7	26,7 ± 5,9	0,254
Albumina sérica (g/dL)	4,3 (4,0–4,6)	4,3 (4,0–4,7)	4,3 (4,2–4,5)	0,775	4,4 (4,0–4,5)	4,3 (4,2–4,7)	0,484
Ácido úrico sérico (mg/dL)	4,6 (3,6–5,9)	4,4 (3,6–5,9)	5,0 (3,8–6,2)	0,389	4,3 (3,6–5,4)	5,0 (3,6–5,1)	0,205
AST (U/L)	19 (15–33)	20 (15–32)	13 (11–18)	**0,039**	16 (14,5–20,5)	24 (16–26)	0,053
ALT (U/L)	20 (16–29)	20 (15–28)	18 (12–24)	0,422	19 (13–26,5)	22 (18–34)	**0,037**
FA (U/L)	70 (55–107)	76 (60–118)	58 (50–67)	0,085	66 (47,5–74,5)	97 (60–138)	**0,011**
HCO_3_ sérico (mmol/L)	25,5 ± 2,7	25,7 ± 3,0	24,5 ± 1,6	0,665	25,6 ± 3,0	25,4 ± 2,5	0,971
Ânion gap sérico (mmol/L)	12,3 ± 4,6	11,9 ± 4,6	13,0 ± 4,1	0,627	11,6 ± 5,0	12,7 ± 4,3	0,429

Abreviações – NA: não aplicável; HbA1C: hemoglobina glicada; 25(OH)D: 25-hidroxi vitamina D; AST: aspartato aminotransferase; ALT: alanina aminotransferase; FA: fosfatase alcalina; HCO3: bicarbonato; AG: ânion gap. Nota – Os dados são relatados como média ± DP, mediana [intervalo interquartil] ou proporções, conforme apropriado.

**Tabela 3:** Exames laboratoriais de urina

	TOTAL (n = 49)	Sem ATRd (n = 41)	ATRd (n = 8)	Valor de “p”	Assintomático (n = 21)	Sintomático (n = 28)	Valor de “p”
Débito urinário (mL/24h)	1687 ± 677	1748 ± 679	1474 ± 658	0,371	1531,1 ± 594,7	1521,2 ± 702,3	0,310
TFG medida (mL/min/1,73m2)	90,5 ± 30,5	93,9 ± 30,6	74,2 ± 29,1	0,125	97,6 ± 22,1	86,1 ± 32,9	0,069
Proteinúria (mg/24h)	141,3 ± 104,2	143,2 ± 106,8	142,4 ± 100,6	0,975	116,8 ± 58,4	151,8 ± 122,5	0,350
Citrato na urina (mg/24h)	396 (271–626)	405 (297–629)	230 (136–566)	0,159	461 (308–691)	329 (257–542)	0,075
HCO_3_ urinário (mmol/L)	0,7 ± 2,1	0,5 ± 1,6	2,0 ± 4,0	0,075	1,1 ± 3,9	0,3 ± 0,7	0,165
Ânion gap urinário (mmol/L)	40,0 ± 47,1	35,9 ± 44,9	62,7 ± 58,5	0,167	45 ± 46,7	29,7 ± 38,3	0,645
EF Na (%)	0,8 (0,6–1,2)	0,7 (0,5–1,1)	1,1 (0,9–1,7)	**0,041**	0,9 (0,6–1,2)	0,9 (0,5–1,0)	0,596
EF K (%)	7,1 (4,7–9,8)	6,9 (4,2–9,4)	7,2 (7–13)	0,286	7,3 (4,1–12,7)	6,9 (4,5–8,7)	0,748
EF Ca (%)	1,7 (1,2–2,4)	1,8 (1,3–2,3)	1,5 (0,9–2,9)	0,546	1,9 (1,6–3,2)	1,6 (1,0–2,0)	0,135
TRF (%)	87,6 (84,3–91,5)	87,6 (83,9–91,5)	88,9 (87,0–90,6)	0,591	87,4 (85,7–91,5)	88,4 (84–91,7)	0,818
EF Mg (%)	3,3 (2,6–5,2)	3,1 (2,5–5,2)	4,6 (3,4–5,7)	0,229	3,8 (2,6–5,7)	2,8 (2,5–4,7)	0,352
EF Ácido úrico (%)	6,7 (5,0–8,8)	6,6 (4,9–8,7)	7,1 (5,8–10,4)	0,546	7,6 (6,2–8,8)	5,7 (4,7–8,3)	0,389
pH urinário (fita reagente)	5,0 (5,0–6,0)	5,0 (5,0–5,5)	6,5 (6,0–6,5)	**<0,001**	5,0 (5,0–5,5)	5,0 (5,0–6,0)	0,199
pH urinário 0h (potenciometria)	5,3 (5,1–5,8)	5,2 (5,0–5,5)	6,2 (5,8–6,5)	**<0,001**	5,2 (5,1–5,4)	5,5 (5,0–5,9)	0,374
NH_4_ urinária 0h (mEq/min/1,73m2)	30,1 (20,8–41,4)	29,1 (20–36,6)	46,4 (30,5–53,6)	**0,016**	29,5 (23,4–38,6)	30,5 (19,9–44,9)	0,944
TA na urina 0h (mEq/min/1,73m2)	21,3 (13,6–26,8)	21,6 (13,3–26,4)	19,0 (15,7–23,6)	0,968	22,4 (14,2–33,4)	18,9 (13,3–23,5)	0,164
ANP (gPtn/kg/24h)	1,3 (1,1–1,8)	1,3 (1,1–1,8)	1,2 (1,1–2,2)	0,203	1,4 (1,0–1,6)	1,3 (1,1–2,4)	0,455
Ingestão de NaCl (g/24h)	6,8 (5,7–11,8)	6,8 (5,8–11,8)	7,2 (3,4–8,6)	0,529	6,7 (5,7–10,4)	6,9 (5,4–11,8)	0,800

Abreviações – EF: excreção fracionada; TRF: taxa de reabsorção de fosfato; ANP: aparência de nitrogênio proteico; pH 0 h = pH antes do protocolo de acidificação urinária; NH4+ 0h = amônia urinária antes do protocolo de acidificação urinária; TA 0 h = acidez titulável antes do protocolo de acidificação urinária. Nota – Os dados são relatados como média ± DP, mediana [intervalo interquartil] ou proporções, conforme apropriado.

A Tabela 4 apresenta dados exclusivamente dos pacientes com ATRd. Um dos pacientes não pôde coletar urina de 24 horas devido à incontinência urinária avançada.

**Tabela 4:** Dados individuais de sujeitos com atrd (n = 8)

	Sujeito #1	Sujeito #2	Sujeito #3	Sujeito #4	Sujeito #5	Sujeito #6	Sujeito #7	Sujeito #8
Sintomático	Sim	Sim	Sim	Sim	Não	Não	Sim	Sim
Transplante hepático	Não	Não	Sim	Não	Não	Não	Não	Não
Uso de Tafamidis	Sim	Não	Não	Não	Não	Não	Sim	Não
HCO3 sérico (mmol/L)	24,9	26,7	23,0	26,0	23,1	22,2	24,9	30,0
TFGe CKD-EPI (mL/min/1,73m2)	120	82	60	100	120	79	94	64
Proteinúria (mg/24h)	104	330	100	150	210	75	28	NA
Citrato na urina (mg/24h)	196	155	77	264	521	699	NA	NA
pH urinário (fita reagente)	6,50	6,00	6,50	6,50	8,00	6,00	6,00	6,00
pH urinário 0h (potenciometria)	6,24	5,83	6,50	6,20	7,10	5,77	5,55	5,75
pH urinário 1h (potenciometria)	6,47	5,69	6,44	6,89	6,29	6,17	5,79	5,72
pH urinário 2h (potenciometria)	5,36	6,12	6,73	6,39	6,22	6,51	6,30	6,11
pH urinário 3h (potenciometria)	6,10	5,96	6,54	5,60	6,64	5,86	5,69	5,80
pH urinário 4h (potenciometria)	6,24	6,11	6,12	6,40	5,90	6,42	6,14	6,56
NH4 urinária 0h (mEq/min/1,73m2)	169	46,4	30,5	**53,6**	45,9	19,5	48	40,3
NH4 urinária 4h (mEq/min/1,73m2)	9,0	45,1	78,4	**65,8**	11,5	62,7	11,5	173,6
TA na urina 0h (mEq/min/1,73m2)	68,3	23,6	15,7	23,5	11,6	19	17,1	14,4
TA na urina 4h (mEq/min/1,73m2)	15,2	18,8	36,2	21,6	16,6	27,4	16,6	8,6

Abreviações – NA: não aplicável; pH 0h: pH antes do protocolo de acidificação urinária; NH4+ 0h = amônia urinária antes do protocolo de acidificação urinária; NH4+ 4h = amônia urinária após protocolo de acidificação urinária, TA 0h: acidez titulável antes do protocolo de acidificação urinária, TA 4h: acidez titulável após o protocolo de acidificação urinária.

A população do estudo foi composta por indivíduos jovens, em sua maioria do sexo feminino, caucasianos, com resultados laboratoriais dentro da faixa normal. 87,7% (n = 44) dos pacientes apresentaram função renal preservada, com creatinina de 0,9 (0,7–1,1) mg/dL e TFGe > 60 mL/min/1,73m^2^. Nenhum apresentou acidose metabólica sistêmica. A maioria apresentava a mutação Val50Met (94%) e havia sido diagnosticada há uma média de 3 anos. Houve uma baixa prevalência de doenças preexistentes de qualquer tipo. Aproximadamente metade dos pacientes era sintomática. Embora 34,7% (n = 17) tenham apresentado proteinúria ≥150 mg na análise da urina de 24 horas, a proteinúria média permaneceu dentro da faixa normal. Somente 8,1% (n = 4) apresentaram albuminúria acima de 30 mg/dia.

Dos 49 pacientes avaliados, 9 apresentaram algum grau de disfunção de acidificação renal: 8 pacientes apresentaram ATRd incompleta (16,3%) e 1 paciente apresentou ATRp (2%). No subgrupo ATRd, 2 pacientes eram assintomáticos.

Não houve diferença significativa entre os subgrupos com e sem ATRd do ponto de vista epidemiológico. No entanto, ao comparar os subgrupos sintomático e assintomático, os indivíduos do subgrupo assintomático eram mais jovens. Os outros três parâmetros com diferença significativa foram: uso de tafamidis, transplante hepático anterior e incontinência urinária, que estiveram presentes somente no grupo sintomático.

Nos resultados laboratoriais, houve uma diferença significativa entre os pacientes com e sem ATRd somente em HbA1c e AST, porém esse fato não parece ter um impacto clínico. Entre os subgrupos sintomático e assintomático, houve diferença nos níveis de potássio, também sem impacto clínico aparente, e nos níveis de ALT e FA, que foram mais elevados no grupo sintomático, provavelmente devido a pacientes com transplante hepático. A proteinúria média do número total de pacientes ficou dentro da faixa normal.

Na análise da urina, encontramos maior EF Na no grupo ATRd. Não houve diferença significativa no citrato urinário entre os grupos com e sem ATRd.

O pH urinário basal foi significativamente mais elevado no grupo ATRd, tanto para as medições com fita reagente quanto potenciométricas, e a amônia urinária basal também foi mais elevada no grupo ATRd. Não houve diferença na acidez titulável basal entre esses dois subgrupos. Na Figura 1, apresentamos a curva de pH urinário de pacientes com ATRd, que não mostra diminuição significativa (pHu <5,3) durante a prova de acidificação. Como mostra também a Figura 1, não houve aumento de uTA ou ${\text{uNH}}_{4}^{+}$ após a prova de acidificação nesse grupo de pacientes com ATRd.

**Figura 1:** (A) Curva de pH urinário por potenciometria no grupo ATRd. (B) Amônia urinária pré (0h) e pós (4h) teste de acidificação urinária no grupo ATRd. (C) Acidez titulável da urina pré (0h) e pós (4h) teste de acidificação urinária no grupo ATRd.

Além disso, obtivemos uma correlação altamente significativa entre o pHU por fita reagente e pHU por potenciometria (Spearman Rho 0,797; p < 0,001), conforme mostrado pela linha de regressão na Figura 2. Com base na curva ROC, o melhor ponto de corte na fita reagente para excluir ATRd foi pHU < 5,5, com sensibilidade de 100% e especificidade de 81%.

**Figura 2:** (A) Curva de correlação linear entre pHU (pH urinário) por fita reagente versus potenciometria. (B) Curva ROC: pH urinário por fita reagente < 5,5 para descartar o diagnóstico de ATRd.

## Discussão

Este estudo transversal incluiu pacientes ambulatoriais com ATTRv acompanhados por uma equipe multidisciplinar em um centro de referência para ATTRv. Os pacientes com ATTRv geralmente apresentam função renal glomerular preservada, um de nossos critérios de inclusão. Encontramos uma prevalência de 18,3% de ATR nessa população, principalmente ATRd, que geralmente é uma condição subclínica e pode preceder o declínio da TFG.

Considerando a amiloidose em geral, existem diversas possibilidades de danos em cada um dos compartimentos renais. Entre os distúrbios tubulares, o mais comum é a ATR tipo 2 como uma manifestação da síndrome de Fanconi na amiloidose AL, que se deve à proteólise prejudicada das cadeias leves no túbulo proximal, comprometendo sua função absortiva^21^. Existem alguns relatos de ATR tipo 4, principalmente na amiloidose AA^22^. Entretanto, considerando apenas a ATTRv, grande parte da literatura sobre envolvimento renal baseia-se em pequenas séries que descrevem principalmente proteinúria e comprometimento da TFG. Nenhum desses estudos relata dados sobre a função tubular.

De acordo com Lobato e Rocha^6^, a ocorrência e o início da nefropatia na ATTRv variam de acordo com a mutação. Em nosso estudo, nos concentramos na mutação V50M, que é a mutação mais comum no Brasil. Nessa mutação, a insuficiência renal geralmente ocorre 5 anos após a microalbuminúria, sendo ainda mais precoce em mulheres^5^. Uma questão crucial na nefropatia por ATTRv é o reconhecimento da presença e distribuição da deposição de amiloide renal^6^. Alguns estudos avaliaram sistematicamente a histopatologia renal na ATTRv Val50Met e mostraram que, além do compartimento glomerular, a medula, a membrana basal dos túbulos distais, as alças de Henle e o interstício são normalmente preenchidos por depósitos amiloides, mesmo nos estágios iniciais da doença^7^. Os depósitos glomerulares são discretos e frequentemente causam albuminúria, o que está bem descrito na literatura. Os pacientes com albuminúria apresentaram um envolvimento amiloide mais extenso do que aqueles sem doença renal clínica^8^. A disfunção renal e o grau de proteinúria se correlacionam com a intensa deposição amiloide em glomérulos, arteríolas e vasos médios, mas não com a deposição em tecidos medulares^6^. Por outro lado, a deposição amiloide está presente no compartimento tubulointersticial mesmo em pacientes com níveis normais de albuminúria^7^.

Portanto, é necessário avaliar se a função tubulointersticial também está prejudicada, uma vez que se demonstrou a presença de depósitos nessa região. Essa pode ser uma ferramenta para diagnóstico precoce da manifestação renal da ATTRv, uma vez que se trata de uma alteração subclínica. A busca por ferramentas de diagnóstico precoce se tornou relevante nos últimos anos devido às novas opções de tratamento que prolongaram a sobrevida dos pacientes. Além disso, a maioria dos tratamentos atualmente disponíveis apenas estabilizam a doença e, portanto, só podem ser iniciados em pacientes que já apresentam algumas manifestações clínicas^2^. Portanto, o diagnóstico precoce é crucial para o tratamento precoce.

Nossa população foi majoritariamente feminina e caucasiana, visto que essa é uma doença tipicamente prevalente em portugueses e seus descendentes. O IMC médio foi normal na população geral e em cada subgrupo, embora o subgrupo sintomático tenha apresentado um IMC mais baixo, porém sem significância estatística. Isso pode ser devido à disautonomia típica com atraso significativo no esvaziamento gástrico e diarreia crônica. Pacientes com transplante hepático ou pacientes em tratamento com Tafamidis foram incluídos apenas no subgrupo sintomático, uma vez que esses são tratamentos utilizados somente em doenças clinicamente manifestas^1^. Para avaliar o possível efeito dos inibidores de calcineurina ou do Tafamidis na função tubular, comparamos os parâmetros clínicos e laboratoriais de pacientes em uso ou não desses medicamentos, sem diferença significativa (dados não apresentados).

Devido à colonização portuguesa no Brasil, a mutação mais comum em nossa coorte foi a Val50Met. A prevalência de doenças pré-existentes potencialmente prejudiciais aos rins foi baixa, o que sugere que os resultados mostrados se devem à nefropatia por ATTRv. A proteinúria e a TFGe estimada pela fórmula CKD-EPI mostraram principalmente uma função renal glomerular preservada em nossa população, o que confirma que nossos achados podem preceder as alterações renais descritas de forma mais clássica.

Apesar da TFG normal de todos os pacientes incluídos, observamos uma elevada prevalência de baixa capacidade de acidificação urinária, principalmente ATRd (16,3%). É interessante notar que nenhum dos pacientes diagnosticados com ATRd apresentou acidose metabólica sistêmica, a chamada forma incompleta de ATRd. A ausência de acidemia pode justificar uma apresentação oligossintomática ou até mesmo assintomática.

A acidose metabólica crônica pode ter efeitos adversos sobre o estado nutricional e o metabolismo ósseo. Embora não tenhamos encontrado acidose metabólica sistêmica, é importante enfatizar que mesmo a forma incompleta de ATRd (sem acidose sistêmica) pode levar à perda óssea e a um aumento do risco de nefrolitíase devido a cálculos de fosfato de cálcio^23^. Tanto os pacientes com acidose tubular renal incompleta quanto os com completa apresentam desmineralização óssea secundária ao tamponamento ósseo. A administração de bicarbonato de potássio a mulheres na menopausa demonstrou resultar em um balanço positivo de cálcio e na melhora da densitometria óssea, que é um indicador indireto do dano osteomineral causado pela acidose metabólica crônica^24^. Além disso, a redução da excreção de ${\text{uNH}}_{4}^{+}$ está associada a um maior risco de DRC em estágio terminal e a um rápido declínio na TFG, sugerindo que a incapacidade de excretar a carga ácida diária é prejudicial ao prognóstico renal^25^. Portanto, é importante diagnosticar precocemente a ATRd para permitir o tratamento precoce e evitar as consequências prejudiciais desse distúrbio.

As tabelas 1, 2 e 3 mostram a comparação entre pacientes sintomáticos versus assintomáticos e pacientes com ATRd versus sem ATRd do ponto de vista epidemiológico, laboratorial e de urinálise. O grupo assintomático apresentou indivíduos mais jovens, provavelmente devido à triagem familiar realizada em familiares de casos confirmados. Embora a hipocalemia seja classicamente descrita na ATRd, não observamos esse distúrbio em nossos pacientes, e não houve diferença significativa nos níveis séricos de potássio entre os grupos com e sem ATRd.

A uTA é a quantidade de H^+^ secretada pelas células do túbulo distal e do duto coletor, ligada aos sais neutros filtrados pelo glomérulo, dando origem aos sais ácidos na urina. A TA não é a única via disponível para a excreção de ácidos^26,27^. Outro mecanismo é a excreção de ${\text{uNH}}_{4}^{+}$ . Ambos permitem a excreção de ácido sem acidez urinária excessiva, com apenas cerca de 1% da carga de H^+^ sendo excretada na forma livre^26,27^. Não houve aumento significativo de ${\text{uNH}}_{4}^{+}$ ou uTA em resposta ao protocolo de acidificação urinária no subgrupo com ATRd.

Embora não tenha havido diferença estatisticamente significativa entre os níveis medianos de citrato urinário dos subgrupos com e sem ATRd, somente o subgrupo com ATRd apresentou um nível considerado hipocitratúrico (<340 mg/24h). Além disso, foi 43,2% menor em comparação com o subgrupo sem ATRd. A falta de significância estatística pode ser devida ao pequeno número de nossa amostra, o que causou uma alta dispersão nessa análise. A hipocitratúria é classicamente associada à ATRd e ajuda a confirmar o diagnóstico. Isso ocorre devido à ávida recuperação de citrato pelas células tubulares proximais no contexto de acidose sistêmica^13^. Shavit e colegas, na revisão de 2016 do protocolo de Walsh de 2007, chegam a sugerir que a combinação de um teste anormal de furosemida e fludrocortisona associado à hipocitratúria e urina alcalina aumenta a probabilidade diagnóstica de ATRd e pode evitar o teste de cloreto de amônio^17^. Além disso, houve uma tendência para que a EF Na fosse ligeiramente maior em indivíduos com ATRd, o que pode exacerbar a hipotensão em pacientes que já apresentam disautonomia. Embora a poliúria tenha sido descrita como um possível sintoma de acidose tubular renal distal, nenhum de nossos pacientes com ATR distal apresentou essa manifestação clínica. Embora houvesse 9 pacientes com transplante hepático, apenas um apresentou ATRd. Sabe-se que os inibidores da calcineurina podem afetar a função tubular, mas em nossa amostra isso foi irrelevante.

Um achado interessante foi a correlação significativa entre o pH urinário medido por fita reagente e por potenciômetro. A fita reagente é útil para a triagem, pois a potenciometria não está amplamente disponível na prática clínica. Portanto, amplia o acesso a uma medição confiável de pHU nesse contexto. Além disso, constatamos que a medição de pHU < 5,5 utilizando uma única fita reagente apresentou uma sensibilidade de 100% e uma especificidade de 93,9% para descartar o diagnóstico de ATRd.

Uma limitação deste estudo foi não termos utilizado sobrecarga de cloreto de amônio^11,28,29^ após o protocolo de furosemida e fludrocortisona para evitar os potenciais efeitos colaterais da acidose metabólica induzida, como náusea, vômito, instabilidade hemodinâmica, etc. Nossos pacientes poderiam já ter disautonomia com tendência à hipotensão e alguns deles apresentavam histórico de transplante hepático, aumentando o risco de efeitos colaterais do uso de cloreto de amônio. Outro problema foi que nossa população era composta em sua maioria por somente um tipo de mutação ou variante genética patogênica (Val50Met). Portanto, os resultados não podem ser extrapolados para outras variantes genéticas.

De acordo com nosso conhecimento, este é o primeiro estudo a demonstrar disfunção tubular renal em pacientes com ATTRv, conforme avaliado pela capacidade de acidificação urinária. Encontramos uma alta prevalência de ATTRv subclínica e sugerimos como diagnosticar essa manifestação renal precoce de ATTRv. O diagnóstico de ATTRv subclínica em pacientes com a mutação Val50Met pode permitir o tratamento específico não apenas para evitar complicações da ATRd, mas também para introduzir novos agentes que estabilizem ou reduzam a produção da TTR mutante. Esses pacientes diagnosticados com ATRd serão acompanhados nos próximos anos. São necessários mais estudos para avaliar o impacto do tratamento medicamentoso na progressão de nefropatias em pacientes com ATTRv.
